# Dosimetric Evaluation of the Effect of Receptor Heterogeneity on the Therapeutic Efficacy of Peptide Receptor Radionuclide Therapy: Correlation with DNA Damage Induction and In Vivo Survival

**DOI:** 10.2967/jnumed.121.262122

**Published:** 2022-01

**Authors:** Giulia Tamborino, Julie Nonnekens, Marijke De Saint-Hubert, Lara Struelens, Danny Feijtel, Marion de Jong, Mark W. Konijnenberg

**Affiliations:** 1 Research in Dosimetric Application, Belgian Nuclear Research Centre, Mol, Belgium;; 2 Department of Radiology and Nuclear Medicine, Erasmus MC, Rotterdam, The Netherlands;; 3 Department of Molecular Genetics, Erasmus MC, Rotterdam, The Netherlands; and; 4 Oncode Institute, Erasmus MC, Rotterdam, The Netherlands

**Keywords:** radiation dosimetry, dose–effect relationship, peptide receptor radionuclide therapy, ^177^Lu-DOTATATE

## Abstract

Our rationale was to build a refined dosimetry model for ^177^Lu-DOTATATE in vivo experiments enabling the correlation of absorbed dose with double-strand break (DSB) induction and cell death. **Methods:** Somatostatin receptor type 2 expression of NCI-H69 xenografted mice, injected with ^177^Lu-DOTATATE, was imaged at 0, 2, 5, and 11 d. This expression was used as input to reconstruct realistic 3-dimensional heterogeneous activity distributions and tissue geometries of both cancer and heathy cells. The resulting volumetric absorbed dose rate distributions were calculated using the GATE (Geant4 Application for Tomographic Emission) Monte Carlo code and compared with homogeneous dose rate distributions. The absorbed dose (0–2 d) on micrometer-scale sections was correlated with DSB induction, measured by γH2AX foci. Moreover, the absorbed dose on larger millimeter-scale sections delivered over the whole treatment (0–14 d) was correlated to the modeled in vivo survival to determine the radiosensitivity parameters α and β for comparison with experimental data (cell death assay, volume response) and external-beam radiotherapy. The DNA-damage repair half-life T_μ_ and proliferation doubling time T_D_ were obtained by fitting the DSB and tumor volume data over time. **Results:** A linear correlation with a slope of 0.0223 DSB/cell mGy^−1^ between the absorbed dose and the number of DSBs per cell has been established. The heterogeneous dose distributions differed significantly from the homogeneous dose distributions, with their corresponding average S values diverging at 11 d by up to 58%. No significant difference between modeled in vivo survival was observed in the first 5 d when using heterogeneous and uniform dose distributions. The radiosensitivity parameter analysis for the in vivo survival correlation indicated that the minimal effective dose rates for cell kill was 13.72 and 7.40 mGy/h, with an α of 0.14 and 0.264 Gy^−1^, respectively, and an α/β of 100 Gy; decreasing the α/β led to a decrease in the minimal effective dose rate for cell kill. Within the linear quadratic model, the best matching in vivo survival correlation (α = 0.1 Gy^−1^, α/β = 100 Gy, T_μ_ = 60 h, T_D_ = 14.5 d) indicated a relative biological effectiveness of 0.4 in comparison to external-beam radiotherapy. **Conclusion:** Our results demonstrated that accurate dosimetric modeling is crucial to establishing dose–response correlations enabling optimization of treatment protocols.

## Visual Abstract

**Figure:**
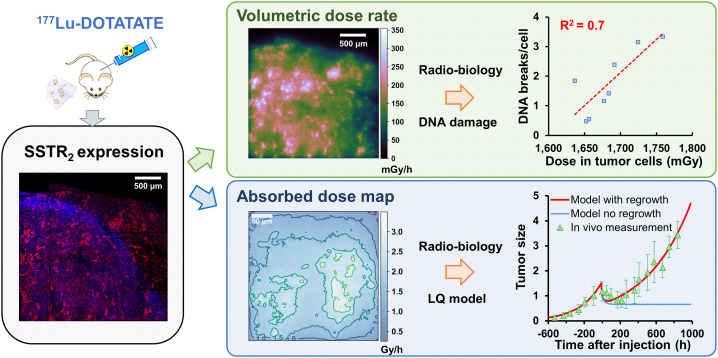

Targeted radionuclide therapy using β-emitting radiolabeled somatostatin analogs is currently applied to patients bearing inoperable neuroendocrine tumors that overexpress the somatostatin receptor type 2 (SSTR_2_) (*1*). Treatment options include ^90^Y-DOTATOC and ^177^Lu-DOTATATE, which is registered as Lutathera (Advanced Accelerator Applications SA).

^177^Lu-DOTATATE therapy has been shown to be successful for many patients, leading to markedly prolonged survival and a better quality of life than with other therapies (*2*,*3*). However, ^177^Lu-DOTATATE therapy is prescribed at a fixed-activity dosing scheme primarily irrespective of the patient’s weight, age, disease burden, uptake, and tumor-specific radiosensitivity (*4*), leading to a suboptimal but overall safe therapy.

In addition, preclinical research into targeted radionuclide therapy has been marked by a scarcity of dosimetric evaluations, sound radiobiologic understanding, and absorbed dose–effect models that could predict tumor response. Nevertheless, evidence strongly implies the existence of an absorbed dose–effect relationship (*5*), which might be used to guide personalized treatment for an optimized therapeutic approach.

Historically, tumor response to targeted radionuclide therapy has been related to macroscopic quantities such as whole-tumor absorbed dose, assuming uniform distribution of the internalized radionuclide and, hence, uniform energy deposition (*6*). However, the biologic response among cells within a tumor can vary greatly, depending on the spatial heterogeneity of dose distributions at multicellular, cellular, and subcellular levels (*7*,*8*). The knowledge of individual cellular absorbed doses and dose rates, together with their radiation sensitivity (α, β), sublethal damage repair, and repopulation capacity, is theoretically indispensable to assess the capability of the treatment to kill every tumor cell, thus impairing tumor regrowth.

At present, few studies have shown that tumor SSTR_2_ expression status can be associated with clinical outcome (*9*,*10*), and a more recent study has addressed the correlation between SSTR_2_ levels and DNA double-strand break (DSB) formation at a preclinical level (*11*). Here, we used SSTR_2_ levels as inputs to model tumor (cancer/healthy cells) and activity heterogeneity on a cellular scale. The resulting absorbed dose and dose rate calculations were used to determine absorbed dose–effect relationships on both a nanoscale (DNA DSBs) and a macroscale (in vivo tumorous cell survival).

## MATERIALS AND METHODS

The biologic experimental data used as input for the dosimetric calculations were part of previous studies performed at Erasmus MC (*11*) and are briefly summarized in the supplemental materials (available at http://jnm.snmjournals.org) (*12*–*17*). Animal experiments were approved by the Animal Welfare Committee of the Erasmus MC and were conducted in accordance with European guidelines.

### Absorbed Dose and Dose Rate Distribution Maps

SSTR_2_ expression of NCI-H69 xenografts from mice injected with ^177^Lu-DOTATATE was assessed by immunofluorescent stainings (*11*). Square tissue sections 3.2 × 3.2 mm in size and with a resolution of 0.625 μm/pixel from 4 independent mice per time point were used to reconstruct 16 voxelized computational models (heterogeneous tumor cell distribution) and the corresponding 16 voxelized sources (heterogeneous radionuclide distribution) at 4 time points (0, 2, 5 and 11 d), as described in the supplemental materials. The input data for the Monte Carlo simulations are represented by 507 × 507 × 289 voxels 5.7 × 5.7 × 10 μm in size.

The Geant4 Application for Tomographic Emission (GATE) Monte Carlo toolkit, version 9.0 (*18*), was used to perform simulations and to score 3-dimensional absorbed dose maps (resolution, 5.7 × 5.7 × 10 μm) within the defined geometry. The average dose was also calculated for tumorous and healthy cells with the DoseByRegion actor (deposited energy per dose voxel mass).

The radioactive source was sampled using the predefined ion source definition (Evaluated Nuclear Structure Data File database), which includes all the spectral components of ^177^Lu. The Livermore physics list (low-energy electromagnetic model) with a production cutoff of 1 μm for the secondary electron was adopted.

The uncertainty when merging the dose maps computed over different cores was calculated according to the method of Chetty et al. (*19*). The total number of particles was chosen to ensure an average error below 6% for all the simulations.

The biodistribution data (*11*) were used to calculate the effective half-life averaged over the whole sections and thus the cumulated activity. The absorbed dose maps were corrected for the number of particles simulated and the bound fraction of activity over different time points, to determine realistic absorbed dose rate distributions over time.

Dose–volume histograms and generalized equivalent uniform dose, as defined in Equation 1, were calculated using a Python (*12*) application to compare the volumetric dose distribution of the heterogeneously distributed radionuclide with the reference case of a uniform spheric source distribution.

The S-value and dose rate distribution calculations for the equivalent uniform spheric phantom were performed on GATE (*18*) using the same physical settings and geometric volume and then compared with OLINDA (*20*) and IDAC-Dose 2.1 (*21*) codes:

$$\text{Generalized equivalent uniform dose}={\left(\frac{1}{N}\;\overset{N}{\underset{i=1}{\sum }}d_{i}^{a}\right)}^{\frac{1}{a}},$$ Eq. 1

where $d_{i}$ represents the absorbed dose in each tumor cell volume (i.e., voxel) and *a* is a negative parameter relating the effects by heterogeneous and uniform dose distributions.

### In Vivo Survival Model

**FIGURE 1.**
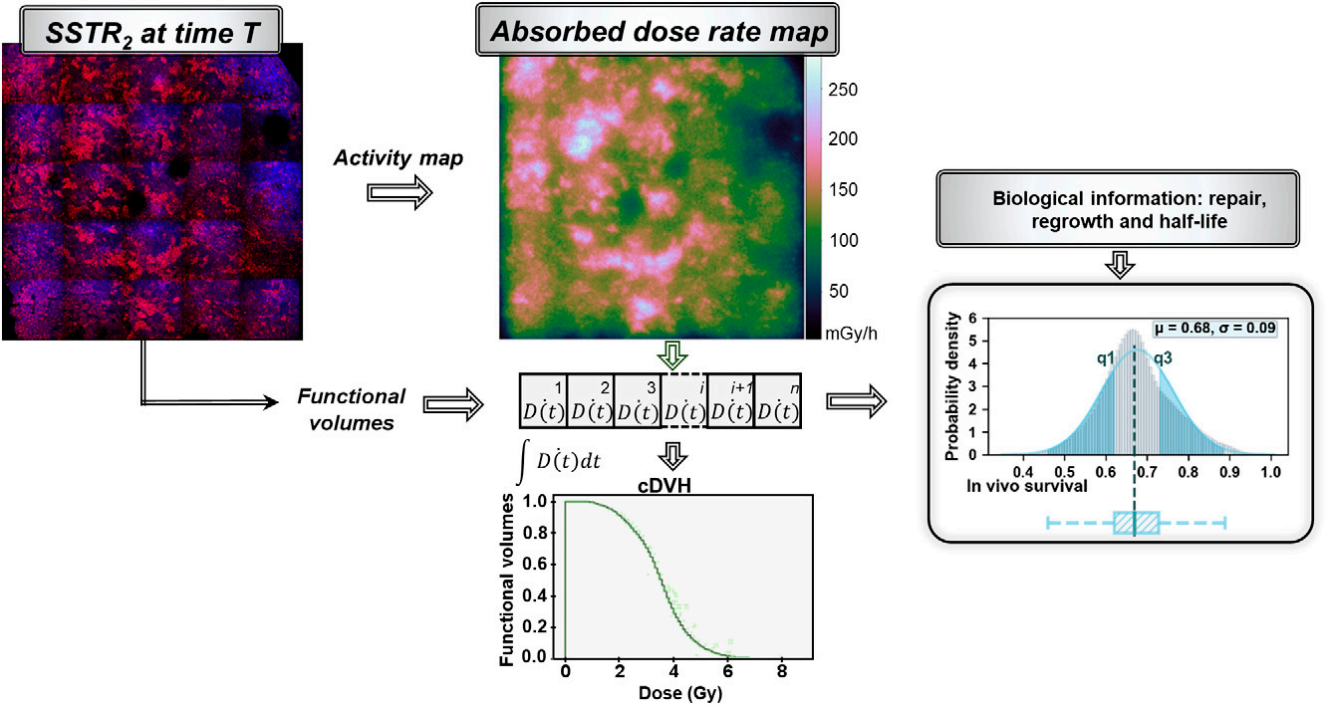
Schematic representation of methodology used to model in vivo survival distribution starting from immunofluorescent SSTR_2_ stainings used to define activity map (source) and functional volumes (tumor cells). Dose rate in each tumor voxel and radiobiologic information are then integrated in linear quadratic model to evaluate in vivo survival distribution within time intervals *E*(*T_i_*). Probability density function of survival (gray histogram) is approximated by gaussian distribution (blue histogram) and reported with box plots to simplify representation. Volumetric absorbed dose computed over tumor cells is alternatively reported in 2 dimensions by means of cumulative dose–volume histograms (cDVH).

The efficacy of the heterogeneous absorbed dose distribution caused by the receptor expression pattern compared with an equivalent homogeneous activity distribution (spheric phantom) was investigated by comparing the corresponding in vivo survivals. Calculations were performed accounting for the dose rate distribution over the tumor cells (i^th^ voxel) at the time of tissue excision, $R_{0}{}^{i}$ (Eq. 2; Fig. 1) or by means of the average dose rate S value determined considering the initial SSTR expression status, hereinafter referred to as the average approach:

$${\frac{dD\left(t\right)}{dt}}^{i}=\left(R_{0}^{i}-P\right)\exp\left(-{\text{λ}}_{e}t\right)+P\;\exp\left(-{\text{λ}}_{p}t\right),\;0\leq t\leq T_{j},$$ Eq. 2

where P (biologic plateau) and ${\text{λ}}_{e}$ (effective half-life) are parameters obtained by fitting the biodistribution as previously reported (*11*),

$$D{\left(t\right)}^{i}=\int_{-\infty }^{\infty }dt\frac{dD{\left(t\right)}^{i}}{dt}=\int_{0}^{T}dt\frac{dD{\left(t\right)}^{i}}{dt}=\left(\frac{R_{0}^{i}-P}{{\text{λ}}_{e}}\right)(1-\exp\;\left(-{\text{λ}}_{e}t\right)\;)+\frac{P}{{\text{λ}}_{p}}\left(1-\exp\;\left(-{\text{λ}}_{p}t\right)\;\right),$$ Eq. 3

G(t)i=2D(t)2[(R0i−P)2(λe2−μ2)(1−e−(λe+μ)t)+(R0i−P)P(λe−μ)(λp+μ)(1−e−(λp+μ)t)+(R0i−P)22λe(μ−λe)(1−e−2λet)−(R0i−P)P(λe−μ)(λp+λe)(1−e−(λp+λe)t)+(R0i−P)P(λp−μ)(λe+μ)(1−e−(λe+μ)t)+P2(λp2−μ2) (1−e−(λp+μ)t)−(R0i−P)P(λp−μ)(λe+λp)(1−e−(λe+λp)t)+P22λp(μ−λp) (1−e−2λpt)], Eq. 4

$$E{\left(t\right)}^{i}=\exp\left(\text{γ}t\right)\;\exp\left(-\text{α}D{\left(t\right)}^{i}-G{\left(t\right)}^{i}\text{β}D{\left(t\right)}^{i^{2}}\right).$$ Eq. 5

The dose *D*(*t*) and the Lea–Catcheside factor *G*(*t*) (supplemental materials), reported in Equations 3 and 4, respectively, are used to describe the in vivo survival *E*(*t*) (Eq. 5), according to the linear quadratic model (*22*). The repair rate μ in Equation 4 was evaluated by fitting the available in vitro γH2AX foci data (Supplemental Fig. 1A). *E*(*t*) was then corrected for tumor repopulation, with repopulation rate γ, obtained by fitting the tumor growth curve according to Equation 6 and imposing *T*_0_ (onset of shrinkage) as equal to 3 d (Supplemental Fig. 1B). The regrowth doubling time (*T_D_*) was then calculated as $\frac{ln(2)}{k_{0}-k_{1}+k_{2}}$, with *k_i_* indicating growth and shrinkage rates and γ = ln(2)/*T_D_*:

$$V=V_{0}\cdot e^{k_{0}t}\cdot max_{t>T_{0}}e^{-k_{1}(t-T_{0})}\cdot max_{t>T_{1}}e^{k_{2}(t-T_{1})}.$$ Eq. 6

The cellular radiosensitivity α and β were taken as variable parameters with an α of 0.264 Gy^−1^, extracted from low-dose-rate (0.002–0.05 Gy/min) external irradiation data (*23*), or an α of 0.14 Gy^−1^, from internal exposure (*24*), and an α/β of 5, 10, and 100 Gy. The effect of a variable radiation sensitivity among the cell population (biologic uncertainty) was tested using the following gaussian distributions: α = 0.264 ± 0.04 Gy^−1^ and 0.14 ± 0.03 Gy^−1^.

The tissue sections excised from 4 different mice at 0, 2, 5, and 11 d were used to calculate the in vivo survival distribution within each time interval *T_i_* (0–2, 2–5, 5–11, and 11–14 d). Then, the final survival distribution was obtained by sampling the average survival distribution in each of the previous time intervals, $E\left(T_{j}-1\right)$, and statistically adding it to the next one as reported in Equation 7:

$$E(T_{j})=E(T_{j}-1)\exp\left(-\text{α}D\left(T_{j}\right)-G\text{β}D{\left(T_{j}\right)}^{2}+\text{γ}T_{j}\right).$$ Eq. 7

The modeled results were then compared with the terminal deoxynucleotidyl transferase–mediated 2′-deoxyuridine, 5′-triphosphate nick-end labeling (TUNEL) assay measurements (*11*) corrected for tumor shrinkage after day 4.

### Correlation Between Absorbed Dose and DSB Level

Using the same methodology outlined earlier in the paper, simulations on smaller tissue sections with a higher resolution (320 × 320 μm with a resolution of 0.325 μm/pixel) costained for γHA2X and SSTR_2_ expression on day 2 were used to seek a correlation with the average absorbed dose delivered to the tumor cells within these 2 d. High-resolution voxelized computational models and sources made of 512 × 512 × 256 voxels with a size of 0.6 × 0.6 × 1.3 μm were used as input for the dose simulations using GATE.

In addition, we identified areas within the large tissue sections (used for the in vivo survival calculations) most likely characterized by a high level of DSB damage using a template-matching technique (Supplemental Fig. 2). High-expression SSTR_2_ cells (with a high level of DSBs) in the smaller tissue sections (used for DSB analysis) were used as a template. The identified areas, expected to present a high level of DSB damage, were then compared with the absorbed dose delivered over 2 d and the dose rate map on day 2. As such, we extended the absorbed dose–to–DSB correlation, found on the small tissue sections, also over larger volumes.

### Statistical Analysis

The curve-fitting result most likely to obtain the input parameters of the in vivo survival model was selected using the corrected Akaike information criterion. Fitting was performed according to the least-squares method, with the Pearson *R*^2^ as the parameter for its goodness (*R*^2^ ≥ 0.7).

The Shapiro–Wilk test was used to analyze whether the DSB data were distributed normally, whereas Q–Q plots verified the normality of dose distributions.

The paired *t* test was used to assess the significance of differences (*P* < 0.05) between sets of data within the S value and the in vivo survival modeling comparison.

## RESULTS

### Good Correlation Exists Between Absorbed Dose and DSBs

**FIGURE 2.**
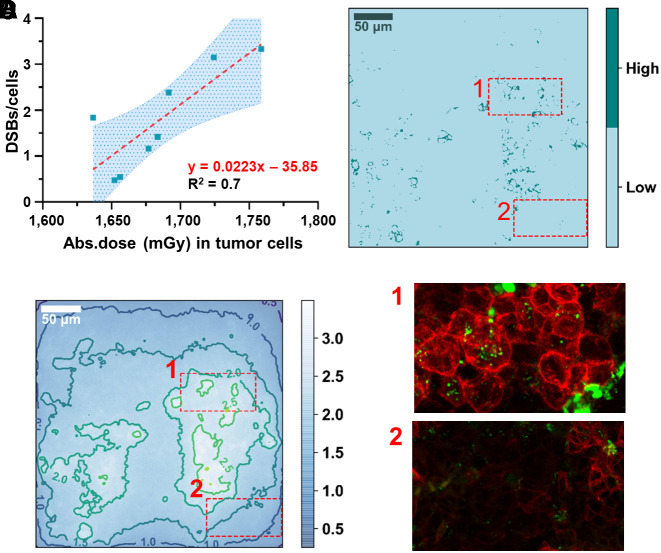
Absorbed dose response. (A) Correlation between average absorbed dose delivered to cancer cells and total number of DSBs measured by γHA2X foci formation. Highlighted area indicates 95% CI. (B) Representative tile-scan of SSTR_2_ stainings thresholded to identify areas of low and high SSTR_2_ expression. (C) Absorbed dose distribution map contoured for isodose levels, with color bar in grays. (D) Zoom of SSTR_2_ (red) and γH2AX (green) immunofluorescent stainings corresponding to high and low levels of SSTR_2_ expression, indicated by 1 and 2, respectively.

The number of DSBs per cell, measured by the total number of γH2AX foci in the smaller costained sections (*n* = 8) taken from the 4 tumors (B1–B4 in Supplemental Table 1), ranged from 0.47 to 3.34 per cell. The absorbed dose to the cancer cells ranged from 1,637 to 1,759 mGy per 30 MBq of ^177^Lu administered. We first fitted the DSBs per cell as a function of the absorbed dose to the cancer cells for each tumor volume separately, verifying a normal distribution for the slopes with the Shapiro–Wilk test (*P* = 0.49). The mean value of the slopes was 0.0235 DSB/cell mGy^−1^. Then, pooling all the data, we found a good correlation, with a slope of 0.0223 ± 0.0231 DSB/cell mGy^−1^ (*R*^2^ = 0.7) (Fig. 2A). For illustrative purposes, the graphical correspondence between SSTR_2_ levels (Fig. 2B), absorbed dose (Fig. 2C), and DSB induction (Fig. 2D) is highlighted for a representative tile-scan image.

The remaining SSTR_2_ expression images and absorbed dose maps, from which the average correlation was drawn, are reported in Supplemental Figure 3.

**FIGURE 3.**
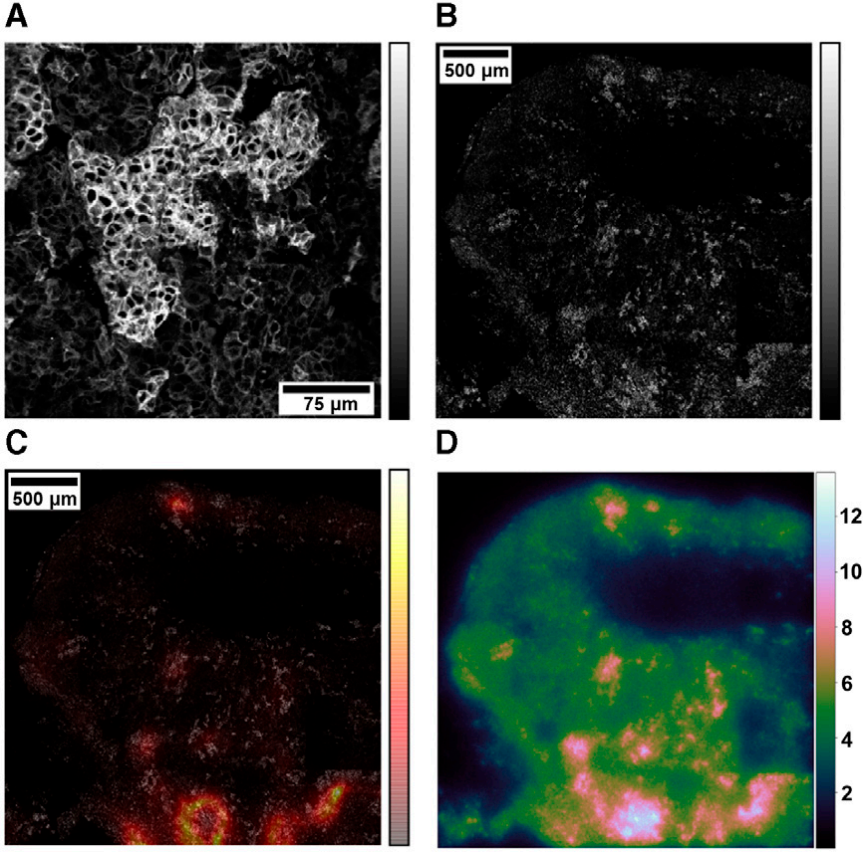
Template-matching technique. (A) Small tissue section used as template. (B) Large tissue section used as test image. (C) Color map indicating similarity score based on χ^2^ value overlaid on top of large tissue section. Color bars indicate pixel intensities of tile scans (grayscale) or similarity map (red-yellow). (D) Absorbed dose map with color bar in grays.

Using the smaller tissue sections characterized by prevalently high-expression SSTR_2_ cells and a high level of DSB induction (Fig. 3A) as a template, we found the location of similar receptor expression patterns in the larger tissue section (Fig. 3B) excised from the same tumor volume (B1–B4) in order to verify the existence of a macroscale correlation. The degree of similarity is indicated by the red-to-yellow color map overlaid on top of the original tissue section image (Fig. 3C). Reporting the corresponding absorbed dose over a 2-d period (Fig. 3D), we observed that the red areas matched the regions with the highest absorbed dose, indicating again a good macroscale correlation with potentially high DSB-forming areas. Similar template-matching results for the 3 remaining tissue samples are reported in Supplemental Figure 4.

### Homogeneous and Heterogeneous Exposures Deliver Comparable Average Absorbed Doses

The average absorbed dose delivered to each tissue section after 2, 5, 11, and 14 d is reported in Supplemental Table 1 in comparison with the corresponding homogeneous spheric exposure. The excised tissue sections were mostly made of tumor cells (94%–100%), similarly to the spheric homogeneous calculations, in which the volume was assumed to be 100% tumorous. Within 2 d, 40% of the dose was delivered to the tumor cells, and the successive time intervals contributed the same percentage (∼20%) to the total absorbed dose.

The homogeneous spheric S value was 8.71E−10, 8.90E−10, and 8.94E−10 Gy/decay using OLINDA, IDAC-Dose 2.1, and GATE, respectively. It differed significantly from the heterogeneous S values, which were 2%–59% higher ($\frac{S_{\mathrm{het}}-S_{\mathrm{hom}}}{S_{\mathrm{hom}}}$) than the homogeneous one. In addition, the heterogeneous S values increased, on average, over time and varied by up to 62%.

The absorbed dose distributions corresponding to the 2 exposure types—reported by means of dose and dose rate maps, frequency dose–volume histograms, cumulative dose–volume histograms, and generalized equivalent uniform doses in Supplemental Figure 5 and Supplemental Table 2—differed significantly from each other, given that only the heterogeneous one was normally distributed, as shown by the corresponding Q–Q plots. The cumulative dose–volume histograms indicated that, on average, 49.17% ± 3.72% of the volume was exposed to a dose equal to or higher than the average dose for the heterogeneous case, compared with 64.46% corresponding to the homogeneous case. Hence, the heterogeneous dose distribution was better represented by its mean value  than   was   the   homogeneous   dose distribution,    in   view   of   its   gaussianlike   behavior.   Indeed ,  the homogeneous absorbed dose distribution over the spheric volume was heavy-tailed and negatively skewed for geometric reasons.

Nevertheless, on average, the absorbed dose characterizing the heterogeneous exposure did not significantly differ from the uniform exposure, diverging prominently only after 5 d.

### Dose Heterogeneity Causes Significant Variation in Treatment Outcome

**FIGURE 4.**
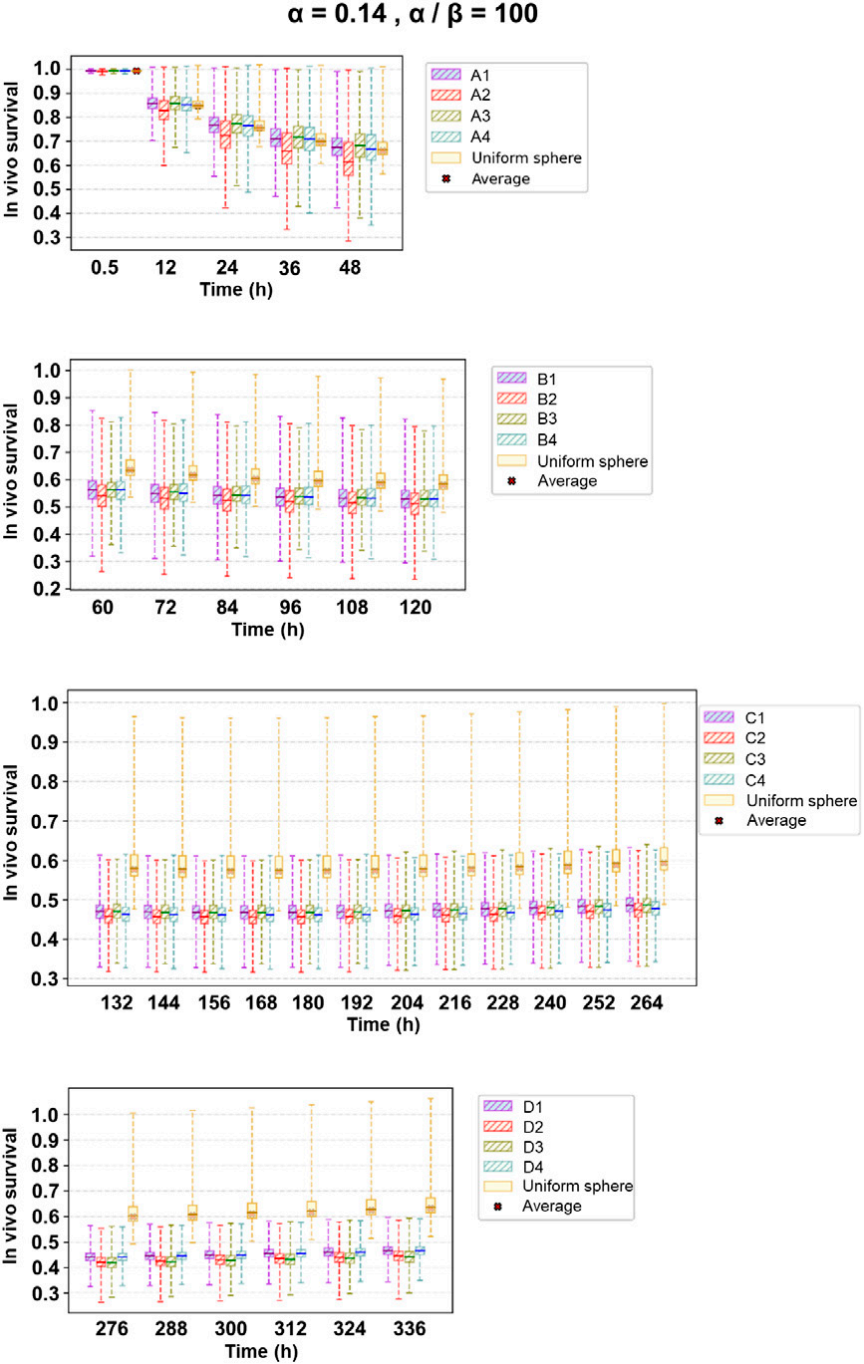
Box plots indicating in vivo survival distribution over time on different excised tissue sections. Whiskers correspond to 1.5 times interquartile range. Corresponding tissue section nomenclature is reported in Supplemental Table 1.

The modeled in vivo survival results corresponding to an α of 0.14 Gy^−1^ (constant), an α/β of 100 Gy, a DNA-damage repair half-life (T_μ_) of 60 h, and a proliferation doubling time (T_D_) of 14.5 d are shown in Figure 4. The box-plot distributions corresponding to the remaining radiosensitivity parameters are reported in Supplemental Figure 6.

No significant difference in survival between the heterogeneous and homogeneous exposures was observed during the first 5 d, when 61% of the radiation dose was delivered. However, in the following days the difference became significant, with the heterogeneous dose delivery becoming more effective (higher cell killing) at preventing tumor regrowth.

Interestingly, the heterogeneous dose rate distribution among the cell population caused a significant dispersion and hence uncertainty in the treatment outcome due to solely physical parameters.

Hypothesizing a gaussian distribution of the radiation sensitivity (α) to account for a realistic tumor heterogeneity caused the SD for cell survival to be so large that the treatment outcome would likely be unpredictable (Supplemental Fig. 7).

**FIGURE 5.**
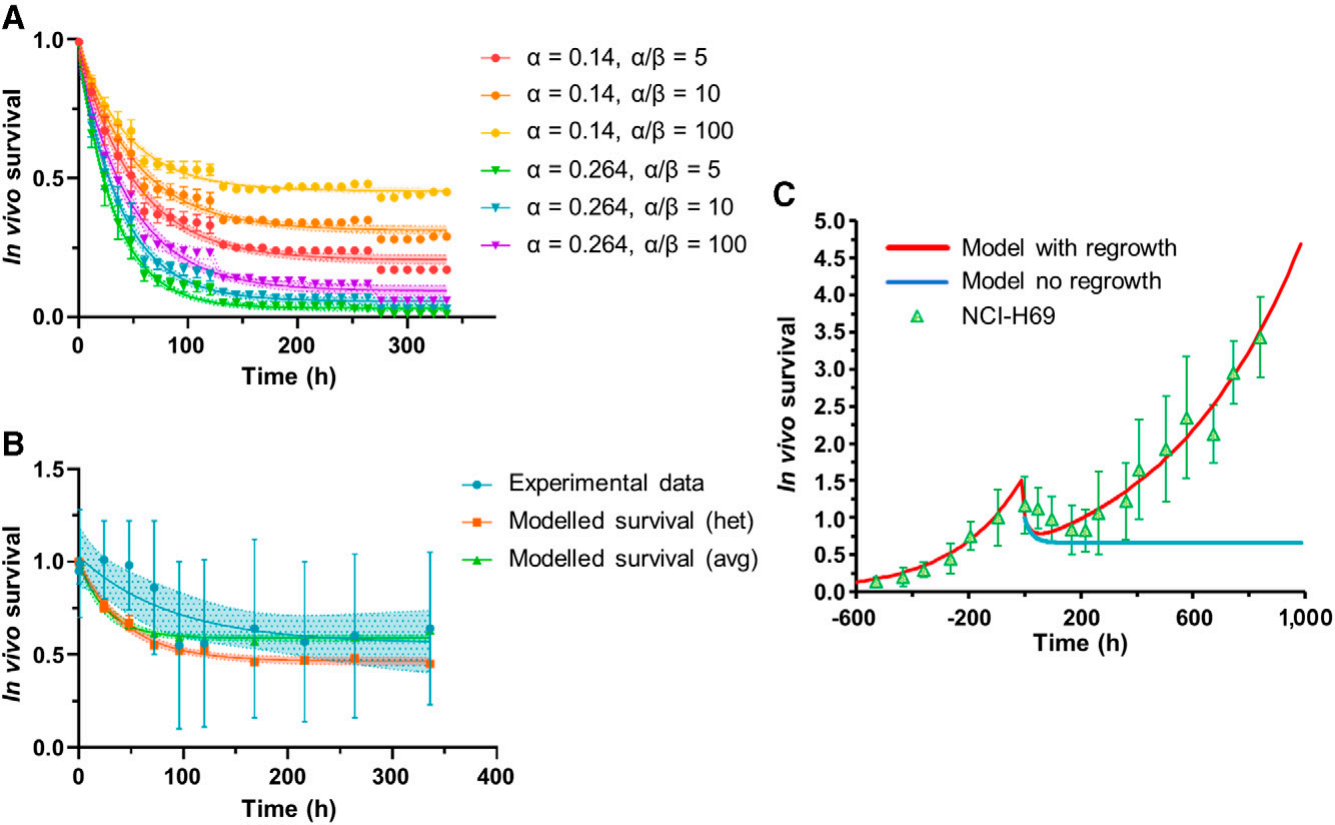
In vivo survival correlations. (A) Radiosensitivity parameter analysis for modeled heterogeneous survival (het). (B) Comparison with experimental data (TUNEL assay; 0 h = time of injection), including modeled results for average approach (avg) (i.e., 1 average S value). α and α/β are reported in Gy-1 and Gy, respectively. Error bars indicate 1 SD. (C) Cell survival correlation with and without regrowth for average calculation approach together with fitted relative tumor volume curve (0 h = onset of shrinkage shifted of 3 d) normalized to volume at time of injection (green triangles).

Averaging the results for the tissue sections belonging to the same time group, we obtained the distribution in Figure 5A, where the constant α and α/β ratios are used as variable parameters. As expected, the higher the α, the greater the cell killing for a given dose, whereas a higher α/β ratio reduced the cell killing by multiple ionization events. Compared with the experimental TUNEL assay results, corrected for the clearance estimated with the tumor growth curve after day 4 (Fig. 5B), the results for an α of 0.14 Gy^−1^ and an α/β of 100 Gy matched well the experimental cell death within the radiobiologic uncertainties.

The in vivo survival correlation calculated with the average approach (α = 0.1 Gy^−1^, α/β = 100 Gy, T_μ_ = 60 h, T_D_ = 14.5 d) was then reported with the experimental tumor volume data, shifting the onset of volume reduction to account for the delay caused by the removal of dead cells (Fig. 5C). The in vivo results corresponding to the heterogeneous exposure (gaussian distributed α) and the uniform exposure are reported in Supplemental Figure 8.

## DISCUSSION

Integrating radiobiologic knowledge into the decision-making process at a clinical level is of the utmost importance in optimizing the therapeutic use of radionuclides. Here, microscale dose assessments based on SSTR_2_ expression patterns from excised tissue sections reveal a good correlation between absorbed dose and DSB induction and a resulting in vivo cell death model that matched the experimental results well.

Recently, it was shown that SSTR_2_ expression levels correlate with DSB induction after ^177^Lu-DOTATATE treatment for NCI-H69 xenografts (*11*). Similarly, a qualitative analysis revealed that ^177^Lu uptake correlates with γH2AX focus induction for CA209478 xenografts (*25*). The same applies at a clinical level, where high SSTR_2_ expression was associated with longer overall and progression-free survival (*9*,*10*). However, in these studies an absorbed dose–DSB correlation, after accurate absorbed dose calculations, was not investigated. Only a few studies have tried to correlate the absorbed dose with DNA damage after ^177^Lu-DOTATATE treatment (*26*,*27*). In this respect, Denoyer et al. (*26*) failed to prove a correlation between the absorbed dose to blood or spleen and the induction of γH2AX foci in peripheral blood lymphocytes of 11 patients undergoing peptide receptor radionuclide therapy, and a poor correlation with bone marrow and tumor was found. Arguably, the reason may lie in the application of general macrodosimetric modeling (MIRD method at an organ level) and, hence, unavailability of specific dosimetry at a functional cell level. Conversely, Eberlein et al. (*27*) found a linear relationship between the number of DSB foci per cell, measured by the colocalized biomarkers γH2AX and 53BP1, and the absorbed dose to the blood. In comparison with our study, we found a 1.5 times higher number of DSB foci per cell per milligray. One reason could be the presence of specific uptake in tumor cells, although the absorbed dose should form an independent parameter. Most probably, the simplified dosimetric modeling causes this difference as well. Indeed, it was demonstrated previously (*28*) that accounting for a realistic distribution of vessel sizes results in absorbed dose estimations lower than the maximum energy deposited by β-particles.

Unlike these studies, our methodology allowed us to investigate the microscale dose distribution over functional volumes (i.e., tumor cells), finding significant differences between homogeneous and heterogeneous dose distributions over the tumor volume. Nonetheless, the heterogeneous dose delivery proved to be as effective as the homogeneous one, possibly because of the long range of ^177^Lu β-particles. In this respect, however, it is important to highlight that the H69 tumor model is most probably more homogeneous in its receptor expression than are actual pancreatic and small-intestine neuroendocrine tumors. Furthermore, the growing interest in short-range radionuclides for targeted radionuclide therapy will increase the impact of heterogeneity as well, making refined dosimetry methods indispensable. For this reason, a thorough investigation into SSTR_2_ expression in 3 dimensions and over time would help further characterize the DNA damage induction.

Accurate dose rate calculation is essential to determine cell death caused by peptide receptor radionuclide therapy as well, since during protracted exposure at relatively low dose rates, induction of DNA lesions competes with DNA damage repair, reducing the cell killing. Our radiosensitivity parameter analysis for the in vivo survival correlation indicated that the minimal effective dose rates for cell kill corresponding to an α/β of 100 Gy are 13.72 and 7.40 mGy/h, respectively, with an α of 0.14 and 0.264 Gy^−1^, respectively. Moreover, a lower α/β leads to a decrease in the minimal effective dose rate for cell kill.

Certainly, besides accurate absorbed dose rate calculations, radiobiologic modeling based on the linear quadratic model requires specific knowledge of the radiosensitivity parameters (α, β, and T_μ_). Our study, in agreement with our previous findings (*29*), demonstrates that extrapolating these parameters from external-beam radiotherapy may not be representative of ^177^Lu-DOTATATE therapy, since they do not account for the intrinsic cellular response to ^177^Lu β-particles. Strikingly, the volume response as a function of time best matched the experimental result, with an α-value of 0.1 Gy^−1^, indicating a relative biological effectiveness of 0.4 in comparison to external-beam radiotherapy (α = 0.264 Gy^−1^). The relative biological effectiveness was derived as indicated for α-particle response (*30*) since the quadratic term could be neglected, despite the long DNA damage repair half-life of 60 h, experimentally determined. Hence, focusing on the difference in radiation sensitivity parameter α between internal and external exposures, our finding resembles the difference reported by Lee et al. (*31*) between ^90^Y and external-beam exposure of DLD-1 colorectal cancer cells (maximal relative biological effectiveness, 0.4).

In addition, our methodology does take into account the potential tumor sensitivity heterogeneity assuming a probabilistic distribution (gaussian) of the α-value, which, combined with the heterogeneous dose rate distribution on a microscale level, could lead to an unpredictable treatment outcome (*32*). However, we did not account for any cell cycle–related change, and such changes might be relevant to include in future models because the fraction of cells in a specific sensitive or radioresistant phase could gradually increase during protracted irradiation (*33*,*34*), leading to a specific radiosensitivity distribution among the cell population. In view of this possibility, sublethal damage repair would vary depending on the dose rate, and the linear quadratic model would not be adequate to describe the tumor response.

More studies investigating the temporal variation in dose rates over time against biologic phenomena such as DNA repair capacity, cell cycle progression,  and  proliferation over the cell population would help to better elucidate the underlying biologic mechanisms of targeted radionuclide therapy to further improve biophysical modeling.

This work was purely a radiobiology modeling study, for which the small cell lung cancer NCI-H69 cell line was the most appropriate choice because, first, it is well established, in contrast to experimental models for gastroenteropancreatic neuroendocrine tumor; second, it is largely used for peptide receptor radionuclide therapy studies (*35*); third, it is classified as a pulmonary neuroendocrine tumor (*36*); and fourth, it expresses neuroendocrine markers, such as chromogranin A, synaptophysin, neuron-specific enolase, protein gene product 9.5, and SSTR_2_, hence demonstrating its neuroendocrine phenotype and overall usefulness as a model for studying SSTR-targeted radionuclide therapy in neuroendocrine tumors (*37*). Approximations and model parameters limit the presented correlation to this specific preclinical setting. Indeed, the higher proliferation rate and homogeneity characterizing available preclinical therapy models may lead to dose overestimations or an incorrect definition of therapy cycles if the results were to be extrapolated to clinics, especially for larger tumor volumes. A further step would be to investigate cell models more representative of neuroendocrine tumors in humans, possibly transplanting them from patients into mice (*38*) and, as such, including such tumor microenvironmental parameters as hypoxia and immune-system effects in order to increase the translational power of biophysical models.

## CONCLUSION

In this study, we developed a methodology to understand and further improve the absorbed dose characterization of peptide receptor radionuclide therapy during in vivo experiments using the SSTR_2_ expression of tumor xenografts. Adopting this methodology, we have established that there is a clear correlation between the absorbed dose and the average number of DSBs per cell after ^177^Lu-DOTATATE exposure. Furthermore, we investigated the radiosensitivity parameters of NCI-H69 cells, concluding that the α-value for cells exposed to ^177^Lu-DOTATATE is significantly different from that of cells exposed to external-beam radiotherapy.

## DISCLOSURE

No potential conflict of interest relevant to this article was reported.

**KEY POINTS**

**QUESTION:** Can dose–effect relationships for DSBs and tumor volume reduction be established for in vivo ^177^Lu-DOTATATE experiments?

**PERTINENT FINDINGS:** Through accurate dosimetric modeling, a good (*R*^2^ = 0.7) linear correlation (slope of 0.022 ± 0.0231 DSB/cell mGy^−1^) between the absorbed dose and the average number of DSBs per cell after ^177^Lu-DOTATATE exposure has been established. Furthermore, the α-value for cells exposed to ^177^Lu-DOTATATE significantly differs from that after external-beam exposure.

**IMPLICATIONS FOR PATIENT CARE:** Distinct differences were found between the cellular dose and the average tumor dose, and these differences might impact clinical tumor dosimetry evaluations for targeted therapy.
